# Severe ischaemic colitis secondary to microvenular thrombosis in a hypercoagulable patient

**DOI:** 10.1093/jscr/rjad721

**Published:** 2024-01-10

**Authors:** Emily R Moran, Anand Trivedi

**Affiliations:** Acute Surgical Unit, Fiona Stanley Hospital, Murdoch, Perth, WA 6150, Australia; Acute Surgical Unit, Fiona Stanley Hospital, Murdoch, Perth, WA 6150, Australia

**Keywords:** ischaemic colitis, microvenular thrombosis, hypercoagulability

## Abstract

A young patient with multifactorial prothrombotic risk factors presented with signs and symptoms of ischaemic colitis, without evidence of bowel hypoperfusion on imaging. She deteriorated with trial of conservative management and required operative management. Intraoperative findings demonstrated severe, confluent large bowel necrosis, sparing the rectum. A total colectomy was performed, with return to intensive care unit due to intraoperative hemodynamic instability. A return to theatre the following day allowed for formation of ileostomy and definitive closure. Histopathological findings of microvenular thrombosis were confirmed. Venous causes of ischaemic colitis present diagnostic challenges due to variable presentation and imaging findings. Microvascular venous thrombosis is likely secondary to multifactorial prothrombotic risk factors including positive autoantibodies and variable compliance with warfarin therapy for aortic value replacement. We present this case of ischemic colitis secondary to an unusual aetiology to emphasize the need to remain clinically suspicious of severe abdominal pain despite relatively benign imaging findings.

## Introduction

Ischaemic colitis typically occurs secondary to impaired blood supply to the colon due to obstructed arterial or venous supply or global hypoperfusion [1]. This leads to ischemia, inflammation, ulceration, and haemorrhage, typically presenting as abdominal pain and rectal bleeding [1]. Venous thrombosis is a less common cause of ischaemic colitis, whereby venous congestion worsens over time to produce an arterial compromise to blood supply [2].

Clinical presentation of ischaemic colitis can be variable and non-specific leading to diagnostic difficulty and delay to surgical input and initiation of management [3]. Diagnosis of ischaemic colitis secondary to microvascular venous thrombosis requires a high index of suspicion in a patient with risk factors for pro-coagulability presenting with signs and symptoms of acute abdomen. We present the case of severe ischemic colitis in a young woman with a complex medical history and multi-factorial hypercoagulability, requiring management with total colectomy.

## Case report

A 37-year-old woman was transferred to the emergency department of a large metropolitan hospital from a rural community health centre with severe abdominal pain, rectal bleeding, and vomiting. She had medical history of mixed connective tissue disorder with a predominant scleroderma phenotype and previously documented positive cardiolipin and beta-2-glycoprotein antibodies. She takes regular mycophenolate, oral steroids, and receives monthly intravenous immunoglobulin infusions. She has an aortic valve replacement which requires anticoagulation with warfarin to target an International Normalised Ratio (INR) of 2.5–3.5, with which she has variable compliance. She lives rurally and is an active smoker.

On examination she was alert and distressed with abdominal pain. She exhibited sinus tachycardia and hypotension on arrival to the emergency department; however she was afebrile and saturating well on room air. Abdominal examination demonstrated a distended, soft, diffusely tender abdomen. New ecchymosis was noted surrounding her umbilicus. A nasogastric tube was *in situ* and had drained 2 l of stomach contents.

Initial management included resuscitation with intravenous fluids while in the emergency department. Haematological investigations were unremarkable aside from a raised lactate of 5.0 mmol/L. INR level at admission was 8.4. A computed tomography (CT) scan of her abdomen and pelvis with intravenous contrast was performed and demonstrated faecal loading with normal bowel wall enhancement.

**Figure 1:**
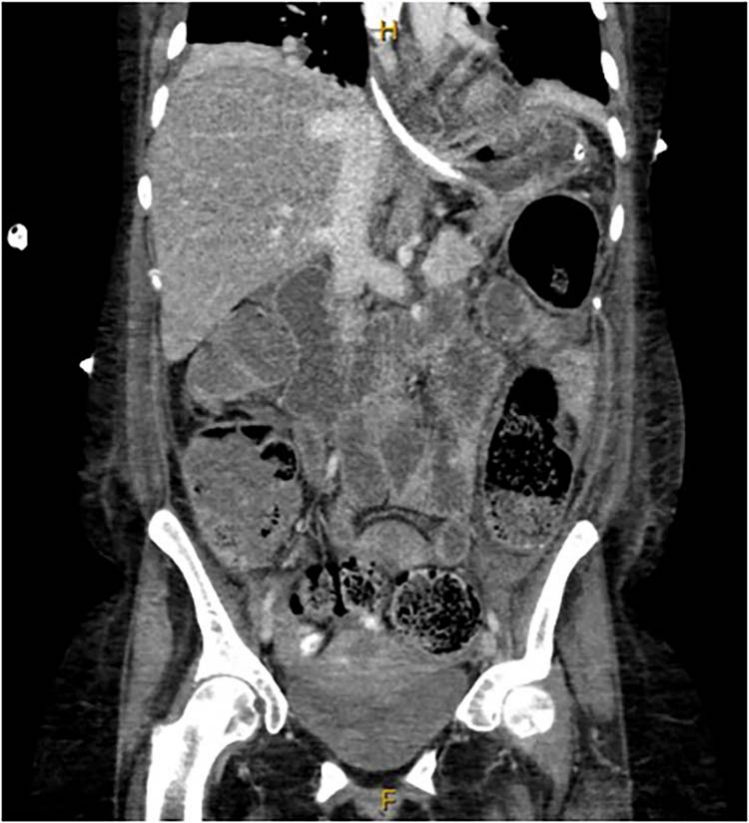
Coronal view of CT abdomen demonstrating normal large bowel wall enhancement with faecal loading.

**Figure 2:**
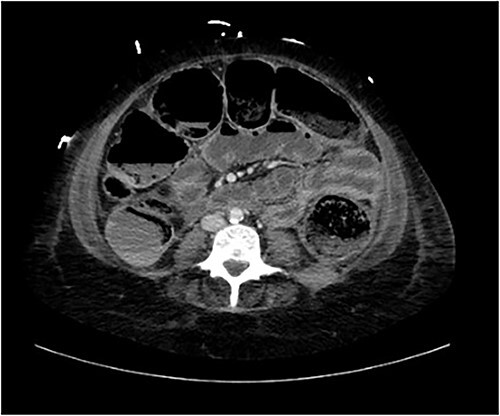
Axial view of CT abdomen demonstrating normal large bowel wall enhancement with faecal loading.

A provisional diagnosis of a ‘bowel obstruction’ was made and she was admitted to hospital for conservative management with nasogastric drainage, intravenous hydration, and aperients. Over the following 24 h, her abdominal pain remained severe and further surgical review was sought. At this stage she exhibited a distended, firm, exquisitely tender abdomen. A repeat CT at this time once again demonstrated faecal loading, normal bowel wall enhancement, and no perforation (Figs 1 and 2). At this stage she was fasted and consented for an exploratory laparotomy under general anaesthesia. She proceeded to theatre after reversal of her INR with prothrombinex and vitamin K.

The operative findings included a grossly distended large bowel with areas of patchy and confluent necrosis, most severe at the splenic flexure and sigmoid colon, sparing the rectum. A total colectomy was performed. This was complicated by haemodynamic instability, requiring temporary closure and admission to intensive care unit (ICU) for stabilization. The following day she returned to theatre for ileostomy formation and definitive closure.

She returned to ICU post-operatively and weaned from sedation. She ceased nasogastric tube feeding Day 3 post-operatively and tolerated oral diet. She made an uneventful further recovery including engaging actively with the stoma education nurses and was discharged from hospital Day 12 post-operatively.

Histopathology confirmed large areas of confluent necrosis most significant at the splenic flexure and sigmoid colon, with patchy venous congestion. Microscopic examination identified combination arterial and veno-occlusive pattern of injury associated with oedema and vascular congestion. This is in keeping with ischaemic colitis secondary to microvenular thrombosis. Thrombophilia testing demonstrated weak positive anticardiolipin IgM and beta-2-glycoprotein IgA antibodies, not meeting laboratory criteria for antiphospholipid syndrome (APS).

## Discussion

Ischaemic colitis has both a variable clinical presentation and varied severity of disease. Abdominal pain and rectal bleeding are most commonly reported [4]; however various clinical manifestations can occur [5, 6]. The onset of pain is slower than that of acute mesenteric ischemia, due to gradual and transient loss of blood supply to the colon secondary to collateral arterial supply [4]. Ischaemic colitis remains an important diagnosis as mortality rates remain as high as 14.9% reported in a recent systematic review [4].

This case report focuses on a case of multi-factorial hypercoagulability as a cause of microvenular thromboembolism, resulting in fulminant ischaemic colitis. Hypercoagulability is risk factor for thromboembolism, and can be due to inherited genetic mutations, acquired prothrombotic states, or secondary to disease such as trauma or sepsis [1].

The risk factors for hypercoagulability noted within this case include presence of APS antibodies and variable warfarin compliance. APS is an acquired thrombophilia mediated by development of autoantibodies [11]. Limited cases of APS-associated severe ischaemic colitis are reported, most in association with diagnosis of catastrophic APS [3, 12–14]. Other reported cases of hypercoagulability and microvascular thrombosis resulting in fulminant ischaemic colitis include that of coronavirus disease 2019 infection [7–9] and inherited Factor V Leiden mutation [10]. In all cases of venous and microvenular ischaemic colitis onset was slow, with a wide variation of primary symptoms and CT imaging findings. Notably, microvascular thrombosis commonly eludes detection on commonly utilized imaging systems such as CT or magnetic resonance imaging [9]. In most reported cases bowel resection was required due to severity of necrosis. In this case, autoantibody levels did not meet the Sydney Criteria [15] for APS; however they may still contribute to microvenular thrombosis risk [9]. A review of the literature did not reveal any cases of ischemic colitis secondary to variable warfarin compliance.

This case illustrates the need for general surgeons to maintain a high index of suspicion of venous causes of ischaemic colitis in patients with prothrombotic risk factors. Previous reports demonstrate that ischaemic colitis secondary to venous micro thrombosis is severe, often requiring total colectomy. Ischaemic colitis can present in variable ways and a clear ischaemic pattern may not be seen on CT imaging, despite florid necrosis at laparotomy.
